# Toward a Monte Carlo approach to selecting climate variables in MaxEnt

**DOI:** 10.1371/journal.pone.0237208

**Published:** 2021-03-03

**Authors:** John L. Schnase, Mark L. Carroll, Roger L. Gill, Glenn S. Tamkin, Jian Li, Savannah L. Strong, Thomas P. Maxwell, Mary E. Aronne, Caleb S. Spradlin

**Affiliations:** Office of Computational and Information Sciences and Technology, NASA Goddard Space Flight Center, Greenbelt, Maryland, United States of America; Texas A&M University, UNITED STATES

## Abstract

MaxEnt is an important aid in understanding the influence of climate change on species distributions. There is growing interest in using IPCC-class global climate model outputs as environmental predictors in this work. These models provide realistic, global representations of the climate system, projections for hundreds of variables (including Essential Climate Variables), and combine observations from an array of satellite, airborne, and *in-situ* sensors. Unfortunately, direct use of this important class of data in MaxEnt modeling has been limited by the large size of climate model output collections and the fact that MaxEnt can only operate on a relatively small set of predictors stored in a computer’s main memory. In this study, we demonstrate the feasibility of a Monte Carlo method that overcomes this limitation by finding a useful subset of predictors in a larger, externally-stored collection of environmental variables in a reasonable amount of time. Our proposed solution takes an ensemble approach wherein many MaxEnt runs, each drawing on a small random subset of variables, converges on a global estimate of the top contributing subset of variables in the larger collection. In preliminary tests, the Monte Carlo approach selected a consistent set of top six variables within 540 runs, with the four most contributory variables of the top six accounting for approximately 93% of overall permutation importance in a final model. These results suggest that a Monte Carlo approach could offer a viable means of screening environmental predictors prior to final model construction that is amenable to parallelization and scalable to very large data sets. This points to the possibility of near-real-time multiprocessor implementations that could enable broader and more exploratory use of global climate model outputs in environmental niche modeling and aid in the discovery of viable predictors.

## Introduction

MaxEnt is one of the most popular software packages in use today by the ecological research community [1–3]. Based on a machine learning approach to maximum entropy modeling, MaxEnt allows researchers to construct ecological niche models (ENMs) that estimate the habitat suitability of a species using occurrence data and a set of environmental variables [1, 2, 4–6]. An abundant literature points to MaxEnt’s effectiveness across a wide range of applications in fields as diverse as biogeography and phylogeny [7], conservation biology and epidemiology [8, 9], invasion biology [10–12], and archaeology [13]. Its merits compared to alternative approaches have been the subject of numerous statistical and methodological analyses, many of which have led to software improvements and refinements to the way MaxEnt is used [14–23]. In this paper, we contribute to this ongoing dialog by describing our efforts to overcome a specific technical limitation of the MaxEnt software that makes the tool difficult to use with large predictor data sets.

In recent years, MaxEnt has become a particularly important aid in understanding the influence of climate change on species distributions [24–29]. The need for reliable climate projections in this work is leading to greater use of global climate model (GCM) outputs as predictors [25]. While creating important new opportunities for research, this trend is also creating a “Big Data” challenge for the MaxEnt community [16]. The largest and most sophisticated GCMs—sometimes referred to as “IPCC-class” models because of the critical role they play in the work of the Intergovernmental Panel on Climate Change (IPCC)—produce petabyte-scale data sets comprising hundreds of variables, a volume that vastly exceeds what is generally used in bioclimatic modeling today [30–32]. Moreover, the direct outputs of these systems are being transformed into derived climate data products on an unprecedented scale [26, 33]. As a result, model tuning and variable selection, which are crucial aspects of any species distribution modeling effort, are becoming more complicated issues [22, 23, 34, 35].

Part of the problem lies in the fact that MaxEnt, like many machine learning systems, acts on its inputs as a piece: predictors and observations must be memory-resident for the program to work [36]. This results in run-times and space requirements that scale linearly with the size of a model’s inputs. In most cases, these scaling properties pose few difficulties. But when the number of predictors under consideration becomes large, compute times can become impractically long, models can become overly complex, and efforts to understand any particular variable’s contribution to model formation, either as an aspect of model analysis or as a way of selecting subsets of variables for further model refinement, can become challenging [17, 34, 37–39]. Clearly, an effective way of dealing with large, externally-stored environmental data sets that preserves the many advantages of MaxEnt while overcoming its current limitations would benefit the MaxEnt community.

In this study, we investigated the potential of an out-of-core Monte Carlo method to help accomplish such an outcome. Monte Carlo optimizations are a common way of finding approximate answers to problems that are solvable in principle but lack a practical means of solution [40]. Out-of-core (or “external memory”) algorithms process data sets that are too large to fit a computer’s main memory [41, 42]. Our objective was to find a useful subset of predictors in a larger collection of environmental variables in a reasonable amount of time. Our proposed solution takes an ensemble approach wherein many MaxEnt runs, each drawing on a small random subset of variables stored in the filesystem, converges on a global estimate of the top contributing subset of variables in the larger collection.

Preliminary results suggest that the method reliably selects a suitable subset of the original predictors that could be explored in more detailed ways and further refined prior to final model construction. Since each model run in the Monte Carlo screening process is independent and uses a set number of variables, the method is totally parallelizable, independent of the intrinsic scaling properties of MaxEnt, and amenable to implementation as an external memory algorithm. If proved to be effective, such an approach could contribute to the ecological modeling process when there is a need to preselect a small set of predictors in a pool comprising a potentially very large number of predictors. This could lead to greater use of climate model outputs by the ecological research community and aid the search for viable predictors when variable selection through ecological reasoning is not apparent.

## Materials and methods

We used Cassin’s Sparrow as a target species in our development efforts. Cassin’s Sparrow (*Peucaea cassinii* Woodhouse, 1852) is an elusive resident of arid shrub grasslands of Middle America and the Southwestern United States [43]. Desert-adapted birds, such as Cassin’s Sparrow, appear to be especially vulnerable to climate change [44, 45]. While the current work does not address a Cassin’s Sparrow science question *per se*, we chose Cassin’s Sparrow as an example of a species whose study could benefit from the technical advances described here. Occurrence data was obtained from the Global Biodiversity Information Facility (GBIF) for the year 2016 [46]. After removing replicates, a total of 1865 records were acquired. To reduce sampling bias and avoid double counting the same individual, we only kept non-overlapping observations within a 16 km buffer, which resulted in a total of 609 observations [47–51].

For predictors, we used Worldclim Version 2.1’s standard (19) Bioclimatic (bioclim) environmental variables at a resolution of 5.0 arc-minutes throughout (Table 1) [52, 53]. These predictor layers were clipped to the coverage area of our observational data, reprojected, and formatted for use with MaxEnt using the Geospatial Data Abstraction Library Version 3.0 (GDAL) software package [54] following the guidelines of Hijmans et al. [55]. We used Variance Inflation Factor analysis to identify collinearities in the predictor data set [56] (S1 Table); however, we did not attempt to minimize collinearity by removing variables, because the current study focuses on stochastic down-selection from a full variable set as a preliminary screening step, which presumably would be followed by refinements such as this prior to final model construction.

**Table 1:** Worldclim bioclimatic variables.

bio01	Annual Mean Temperature
bio02	Mean Diurnal Range (Mean of monthly (max temp—min temp))
bio03	Isothermality (BIO2/BIO7) (×100)
bio04	Temperature Seasonality (standard deviation ×100)
bio05	Max Temperature of Warmest Month
bio06	Min Temperature of Coldest Month
bio07	Temperature Annual Range (BIO5-BIO6)
bio08	Mean Temperature of Wettest Quarter
bio09	Mean Temperature of Driest Quarter
bio10	Mean Temperature of Warmest Quarter
bio11	Mean Temperature of Coldest Quarter
bio12	Annual Precipitation
bio13	Precipitation of Wettest Month
bio14	Precipitation of Driest Month
bio15	Precipitation Seasonality (Coefficient of Variation)
bio16	Precipitation of Wettest Quarter
bio17	Precipitation of Driest Quarter
bio18	Precipitation of Warmest Quarter
bio19	Precipitation of Coldest Quarter

In addition to GDAL, our computing environment comprised MaxEnt Version 3.4.1 [57], R Version 4.0.1 [58], the ENMeval Version 0.3.0 R package [59], RStudio Version 1.2.5033 [60], and ENMTools Version 1.4.4 [61] running on a 2.8 GHz Intel Quad-Core i7 MacBook Pro with 16 GB of memory.

One of the most common uses for ecological niche models is to identify important variables [62, 63]. In this study, we used MaxEnt in two different ways to find the six most influential range- and niche-defining bioclim variables for Cassin’s Sparrow. The choice of “top six” for this evaluation was based on our experience that six or fewer predictors generally predominate in such models.

First, we developed a baseline model using the stand-alone MaxEnt program operated through its graphical user interface (GUI). MaxEnt users can apply various combinations of five mathematical transformations (‘feature classes’ or FCs) to predictor variables to enable more complex fits to the observational data. The available feature types for continuous variables are linear (L), quadratic (Q), hinge (H), product (P), and threshold (T) [4]. Users can also adjust a regularization multiplier (RM) to maximize predictive accuracy and offset the overfitting that FC adjustments can introduce. By default, MaxEnt uses the LQHP feature classes and a regularization multiplier of 1.0 when there are more than 80 training samples, which was the case here [57]. We confirmed the appropriateness of these settings for our data by performing a comprehensive ENMeval scan of all five FC classes across RMs ranging from 0.5 to 4.0 in half-step intervals [38, 59] (S2 Table). We applied MaxEnt’s default FC and RM settings (i.e. the “Auto features” setting) with 10 replicate cross-validation and jackknife evaluation of variable importance. Ten thousand background points were selected from across the study area following the recommendations of Phillips et al. [64] and Fourcade et al. [48].

MaxEnt provides three algorithm-specific indicators of variable importance: percent contribution, permutation importance, and change-in-gain based on jackknife analysis of individual variables [3, 62, 65]. No single measure is sufficient to identify which variables are best for producing a final model [23]; however, for screening purposes and to simplify comparisons in this initial evaluation, we used permutation importance as our sole indicator of variable importance. We determined the average permutation importance for each variable in three replicated runs. The top six predictors in the three-run ensemble constituted our preselected variable set. These were then used to develop a final MaxEnt baseline model.

We then developed an alternative method to identify the top six variables using a random selection of variables to produce sets of predictors for repeated MaxEnt runs. We implemented our Monte Carlo approach as an R script that invokes MaxEnt through ENMeval, which provides convenient control over model settings, built-in evaluation metrics, and improved performance [38, 59]. To reduce variability and isolate outcomes as much as possible to the effects of the sampling process, we again used MaxEnt’s default feature class setting of LQHP and a regularization multiplier setting of 1.0 as fixed parameters in all the Monte Carlo runs. We defined ensemble, in this case, to mean a collection of 100 sprints, where each sprint consisted of ten runs. A tally table was used to maintain a count of the number of times a variable was used in a run along with a cumulative sum of the variable’s permutation importance. The tally table thus provided the information needed to determine the average permutation importance of a predictor at any point along the way.

To process a sprint, we initialized each of its ten model runs with a random subset of environmental variables read from the filesystem. Random integers drawn from a uniform distribution ranging 1–19 corresponding to the 19 bioclim predictors were used to make the selection. At the conclusion of each run, the tally table was updated appropriately. At the conclusion of each sprint, we computed a MaxEnt model using the six predictors in the original starting set having the highest average permutation importance values at that point. This process was repeated 100 times to produce a complete ensemble. This resulted in an evolving progression of models that converged on a stable assemblage of top six predictors over the course of an ensemble. We assessed the algorithm’s performance in two ensembles. In the first, we chose two random variables for each sprint run; in the second, six random variables were used for each run. This resulted in an overall total of 2000 MaxEnt runs.

Given our focus on variable screening as an initial step in the modeling process, we did not perform a comprehensive analysis on any of the six-variable final models. We did, however, look at several attributes of these models to gain a general understanding of how the Monte Carlo algorithm was performing. The predictive distribution maps produced by the models were judged for reasonableness based on first-hand knowledge of the species, its habitat preferences, and known range [50]. We further compared these predictions to observational records from Cornell Lab’s eBird citizen-scientist database [66]. We used the area under the operating curve (AUC) [67] as an indication of a model’s classification accuracy (higher values indicating greater accuracy) and the Akaike information criterion corrected for small sample size (AICc) [68] as a measure of relative explanatory power (lower values indicating less information loss). Model similarity was compared with Warren’s I-statistic [69] and Schoener’s D statistic [70] (higher values in both indicating greater similarity) using ENMTools. Single-processor run times were recorded to aid our understanding of algorithm performance and help identify opportunities for multiprocessor parallelization. Input data and the R script used in the study are provided as S1 File.

## Results

**Fig 1:**
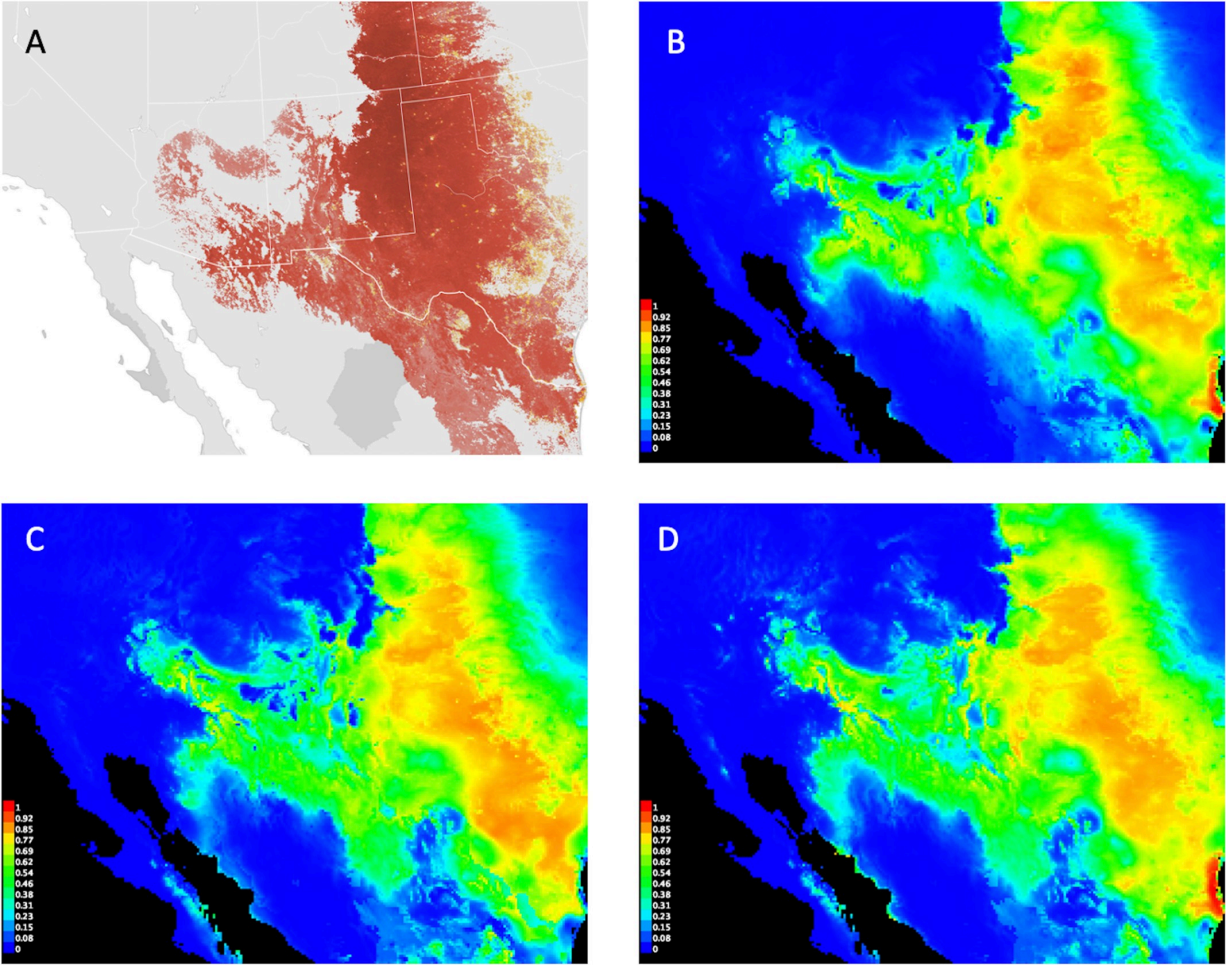
Cassin’s Sparrow distribution maps. Cassin’s Sparrow range map (A) compared to the species’ predicted habitat suitability distributions obtained from the MaxEnt baseline (B), Monte Carlo Ensemble #1 (C), and Monte Carlo Ensemble #2 (D). Image (A) provided by eBird (www.ebird.org), created 28 July 2020, and reprinted from [71] under a CC BY license, with permission from the Cornell Lab of Ornithology. Images (B)–(D) created by the authors show MaxEnt logistic output, which can be interpreted as an estimated probability of presence between 0 and 1 with warmer colors indicating better predicted conditions [72].

On the basis of permutation importance, 13 of the 19 original bioclim variables were among the top ten most contributory predictors across all three replicated runs of the MaxEnt baseline: bio02, bio03, bio05, bio06, bio08–bio12, bio14, bio15, bio17, and bio18 (Table 2). Of those, bio02, bio05, and bio14 appeared in only one run each at 10th place. Bio18 showed strong dominance throughout. When performance was averaged across all three runs, the top six contributory variables in the ensemble collectively accounted for 65% of overall permutation importance (ensemble average). In descending order of importance, the top six predictors included bio18, bio03, bio10, bio15, bio11, and bio06. When these six top-contributing variables were used in a final MaxEnt run, the model’s four most contributory variables (bio18, bio03, bio10, and bio15) accounted for approximately 86% of overall permutation importance, and its predicted habitat suitability distribution corresponded well with what is known about the natural history of the species and observational records for Cassin’s Sparrow for the year 2016 (Fig 1) [66].

**Table 2:** Results of MaxEnt baseline and Monte Carlo selection trials.

	Bioclim Environmental Variables Permutation Importance																					Permutation Importance		Total Run	Total Run Time		Avg Random
MODELS	bio01	bio02	bio03	bio04	bio05	bio06	bio07	bio08	bio09	bio10	bio11	bio12	bio13	bio14	bio15	bio16	bio17	bio18	bio19	AUC	AICc	% Top 3	% Top 4	Count	Mins	Hrs	Samples / Variable
Maxent Baseline																											
Run #1	1.0	4.3	5.6	2.8	0.6	0.9	0.9	5.2	4.2	8.4	4.9	4.6	1.0	2.7	8.3	0.0	2.2	30.6	1.7					1	151	2.5	–
Run #2	1.7	2.9	6.4	1.9	3.5	9.9	0.5	3.5	3.9	5.4	7.7	4.8	1.0	3.5	8.3	0.8	0.5	30.1	3.6					1	120	2.0	–
Run #3	0.8	3.4	6.1	1.3	1.9	10.4	1.0	2.5	4.5	7.6	8.3	4.6	2.3	3.4	7.1	0.1	3.4	28.9	2.5					1	100	1.7	–
Ensemble avg	1.2	3.5	6.0	2.0	2.0	7.1	0.8	3.7	4.2	7.1	7.0	4.7	1.4	3.2	7.9	0.3	2.0	29.9	2.6								
Final model			14.1			6.6				13.8	7.3				10.9			47.4		0.818	12,222	75.3	86.2	1	18.0	0.3	–
Monte Carlo Selection																											
Ensemble #1	*(two random variables per sprint run)*																										
Sprint 025					17.6			10.8	9.3				22.2			14.3		25.9		0.801	12,229	65.7	80.0	250	181	3.0	26
Sprint 050					15.1			11.7	11.2				21.2			15.2		25.5		0.802	12,231	61.9	77.0	500	355	5.9	53
Sprint 100				24.1	18.2			6.2					7.6			4.1		39.7		0.806	12,252	82.0	89.6	1000	710	11.8	105
Ensemble #2	*(six random variables per sprint run)*																										
Sprint 025			25.4			3.6					15.6		7.7			3.8		43.9		0.801	12,376	84.9	92.6	250	438	7.3	79
Sprint 050			26.6			4.9					13.5		3.8			3.7		47.7		0.807	12,168	87.8	92.7	500	856	14.3	158
Sprint 100			26.5			5.5					18.9		4.1			2.6		42.4		0.805	12,152	87.8	93.3	1000	1793	29.9	316

**Fig 2:**
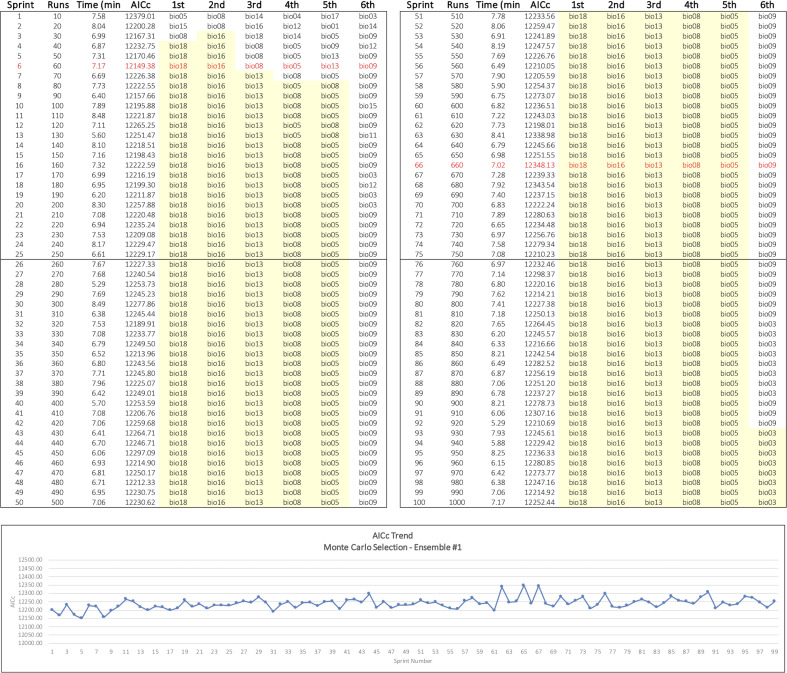
Monte Carlo Ensemble #1 results. Two random variables at a time were chosen for each MaxEnt sprint run. The sprint log on top shows the progressive selection of a stable set of top six variables in yellow. The graph on the bottom shows the narrow range of fluctuating AICc values over the course of the ensemble runs. Maximum and minimum AICc values are shown in red.

**Fig 3:**
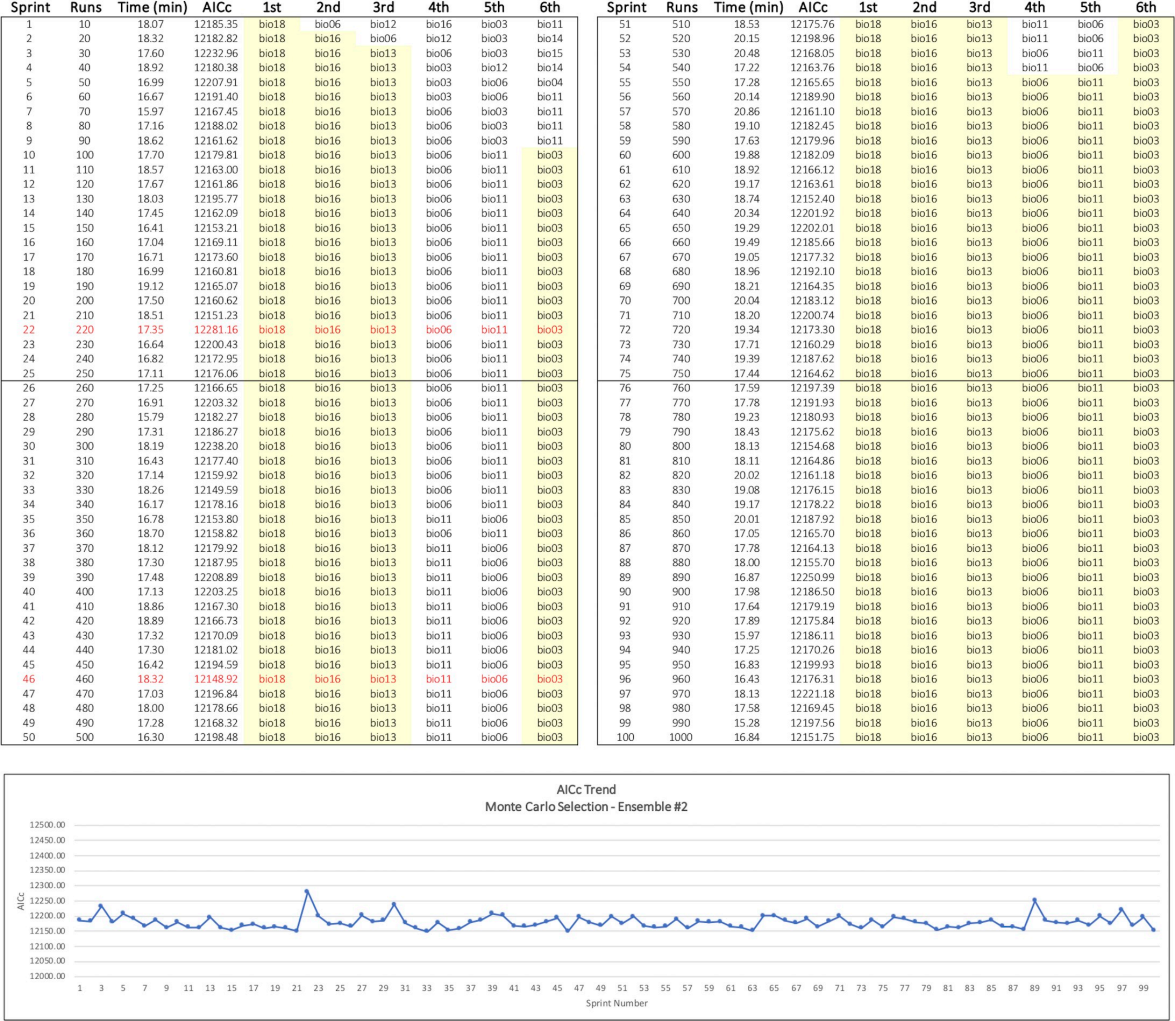
Monte Carlo Ensemble #2 results. Six random variables at a time were chosen for each MaxEnt sprint run. The sprint log on top shows the progressive selection of a stable set of top six variables in yellow. The graph on the bottom shows the narrow range of fluctuating AICc values over the course of the ensemble runs. Maximum and minimum AICc values are shown in red.

A distinct pattern of progression toward a stable subset of key variables was observed in the Monte Carlo ensembles (Figs 2 and 3). In both cases, the top three contributory variables among the top six were selected early in the sprint runs, and AICc values fluctuated within a narrow range around an average that changed little over the course of the selection process. Greater variability in the composition of the top six subset was seen in Ensemble #1 where two random variables at a time were selected for each sprint run (Table 2 and Fig 2). In Ensemble #2, where six random variables at a time were selected for the MaxEnt runs, the top six variables were identified by the 25th sprint and had settled into their final rank order by sprint 54 (Fig 3). Ensemble #2 appeared to produce the best overall results and shared four variables in common with the top six selected by the MaxEnt baseline (bio18, bio03, bio11, and bio06) (Table 2). Ensemble #2’s final model had the lowest overall AICc, and its four most contributory variables accounted for approximately 93% of overall permutation importance, the highest attained overall.

Ensemble #1 had only one variable in common with the top six selected by both the baseline run and Ensemble #2. What accounts for this difference is not immediately apparent; however, we speculate that the random pair-wise comparisons occurring in Ensemble #1 may alter the relative global influence of the collinearities known to exist in the bioclim variables [73–75]. The average number of times a variable was sampled appeared to have a marginal, positive influence on resulting model quality once an adequate minimum was attained. Ensemble #2 results suggest that at least 80 uniformly distributed samples per starting-set variable are needed to identify a reasonable top six set of variables; the best overall model resulted from over 300 samples per variable (Table 2).

## Discussion

The most striking outcome of the study is the Monte Carlo method’s ability to select a set of top-contributing predictors by randomly sampling a collection of variables that is comparable to the top-contributing predictors identified by MaxEnt when it operates on the collection as a whole (Table 2). The top six selected predictors in the MaxEnt baseline and the Monte Carlo ensembles produced predicted habitat suitability distributions that are nearly indistinguishable from one another (Fig 1). The two approaches each identified four variables that collectively contributed more than 80% to the formulation of their respective models. And across the board, models based on selected predictors showed a high degree of similarity in Schoener’s D and the I-statistic (Table 3). This gives us confidence that the Monte Carlo method will be able to preselect viable predictors when applied to a larger variable pool.

**Table 3:** Model similarity metrics.

Schoener’s D Statistic										
MODELS ↓ →	Maxent-Run1	Maxent-Run2	Maxent-Run3	Maxent-Final	MC-E1-025	MC-E1-050	MC-E1-100	MC-E2-025	MC-E2-050	MC-E2-100
Maxent-Run1	1	0.9712	0.9738	0.9355	0.8629	0.8630	0.9044	0.8882	0.8903	0.8906
Maxent-Run2	x	1	0.9793	0.9397	0.8639	0.8648	0.9078	0.8915	0.8949	0.8947
Maxent-Run3	x	x	1	0.9354	0.8579	0.8586	0.9039	0.8871	0.8903	0.8902
Maxent-Final	x	x	x	1	0.8667	0.8673	0.9214	0.9104	0.9138	0.9142
MC-E1-025	x	x	x	x	1	0.9880	0.9006	0.8810	0.8801	0.8785
MC-E1-050	x	x	x	x	x	1	0.9026	0.8813	0.8804	0.8786
MC-E1-100	x	x	x	x	x	x	1	0.9393	0.9389	0.9365
MC-E2-025	x	x	x	x	x	x	x	1	0.9815	0.9810
MC-E2-050	x	x	x	x	x	x	x	x	1	0.9844
MC-E2-100	x	x	x	x	x	x	x	x	x	1
Warren’s I Statistic										
MODELS ↓ →	Maxent-Run1	Maxent-Run2	Maxent-Run3	Maxent-Final	MC-E1-025	MC-E1-050	MC-E1-100	MC-E2-050	MC-E2-100	MC-E2-100
Maxent-Run1	1	0.9991	0.9994	0.9948	0.9760	0.9758	0.9874	0.9828	0.9833	0.9834
Maxent-Run2	x	1	0.9995	0.9949	0.9760	0.9760	0.9878	0.9831	0.9838	0.9839
Maxent-Run3	x	x	1	0.9945	0.9748	0.9748	0.9869	0.9823	0.9830	0.9831
Maxent-Final	x	x	x	1	0.9755	0.9753	0.9894	0.9871	0.9878	0.9880
MC-E1-025	x	x	x	x	1	0.9998	0.9887	0.9798	0.9797	0.9784
MC-E1-050	x	x	x	x	x	1	0.9889	0.9795	0.9794	0.9781
MC-E1-100	x	x	x	x	x	x	1	0.9910	0.9912	0.9906
MC-E2-025	x	x	x	x	x	x	x	1	0.9996	0.9994
MC-E2-050	x	x	x	x	x	x	x	x	1	0.9996
MC-E2-100	x	x	x	x	x	x	x	x	x	1

We note that among the top six variables resulting from all the MaxEnt runs, only two (bio06 and bio16) present collinearity issues with respect to the other selected variables: bio06/bio11, bio16/bio13, and bio16/bio18 are potentially problematic pairs. (Tables 2 and S1). However, there were no collinearity issues among the top four variables in any of the runs, and the top four selected by the Monte Carlo method contributed significantly to their models, with permutation importance ranging from 77% to 93% (Table 2). When used as an initial screening step, it would be crucial, at this point, for modelers to perform other quality control steps prior to final model construction.

While perfecting an ENM for Cassin’s Sparrow was not a goal in this study, we also note that the selected variables have biological relevance. In particular, the three temperature-derived variables, bio03 (isothermality), bio06 (minimum temperature of coldest month), and bio11 (mean temperature of coldest quarter), and the precipitation-derived bio18 (precipitation of warmest quarter) have been identified as important influences on the distribution of arid-adapted birds in general and Cassin’s Sparrow in particular [50, 76–79].

The most significant drawback identified in the study was the long run times. MaxEnt’s linear scaling behavior can be challenging in a single-processor environment. In the baseline runs, producing a single model through MaxEnt’s GUI using our selected settings involved writing many files to disk and took from 18 minutes (with six variables) to over two hours (with all 19 variables). MaxEnt in the R environment outputs memory-resident objects, which results in faster run times. Still, with its repeated invocations of MaxEnt, Ensemble #2 took nearly 30 hours to complete (Table 2). This too is a result of a linear scaling property; however, the Monte Carlo method’s scaling behavior is not determined by the MaxEnt program, since each of the MaxEnt runs in the Monte Carlo method operates on a set number of predictors. The method’s linear scaling property is determined, instead, by the need to adequately sample the starting set of environmental variables in order to obtain a good result.

This is an important distinction. It means that each of the Monte Carlo MaxEnt runs is entirely independent from all other runs in the ensemble. This high level of subtask independence is sometimes referred to as an “embarrassingly parallel” workload. It makes practical, multiprocessor implementations of the method possible. If 1000 processors were recruited into service—which is becoming increasingly convenient with the proliferation of multiprocessor, high-performance cloud computing—a 1000-run ensemble could conceivably take as long as a single MaxEnt run.

The potential significance of this advantage becomes apparent when one considers the method’s use with large collections of environmental data. The Monte Carlo approach described here provides an approximate solution to the problem of finding a useful *k*-size subset of an *n*-size collection of variables. In principle, there are *n*! / [*k*!(*n*-*k*)!] variable combinations to consider in such an evaluation, a staggering 27,000-plus six-variable subsets with the 19 bioclim variables alone. Algorithms that accomplish variable selection through stepwise removal or are otherwise bound to the linear scaling properties of underlying software components are inherently unable to exhaustively explore this combinatorial space. A Monte Carlo method makes such a search possible by randomly sampling the universe of possible combinations and returning approximate solutions in practical amounts of time, particularly if implemented as a high-performance cloud service.

While these findings are preliminary, they address an important issue facing the modeling community. There is heightened awareness of the significance of dimensionality in understanding environmental spaces and the importance of variable selection in modeling those spaces [23, 34, 80]. This awareness is accompanied by a recognition that logistic difficulties often preclude examining large numbers of variables [62]. This has led to a search for alternative means of variable selection and calls for process automation [22, 23, 62, 78, 81]. A comprehensive review of these approaches is beyond the scope of this paper; however, it is worth nothing that even among the most recent work in this area, many of the solutions put forward—such as manual prescreening for collinear variables, greater use of biological insight in variable selection, broader use of memory-resident machine language-based analysis software, etc.—do not, in general, scale well. They are unlikely to accommodate the petabyte and even larger size data collections on the horizon.

**Fig 4:**
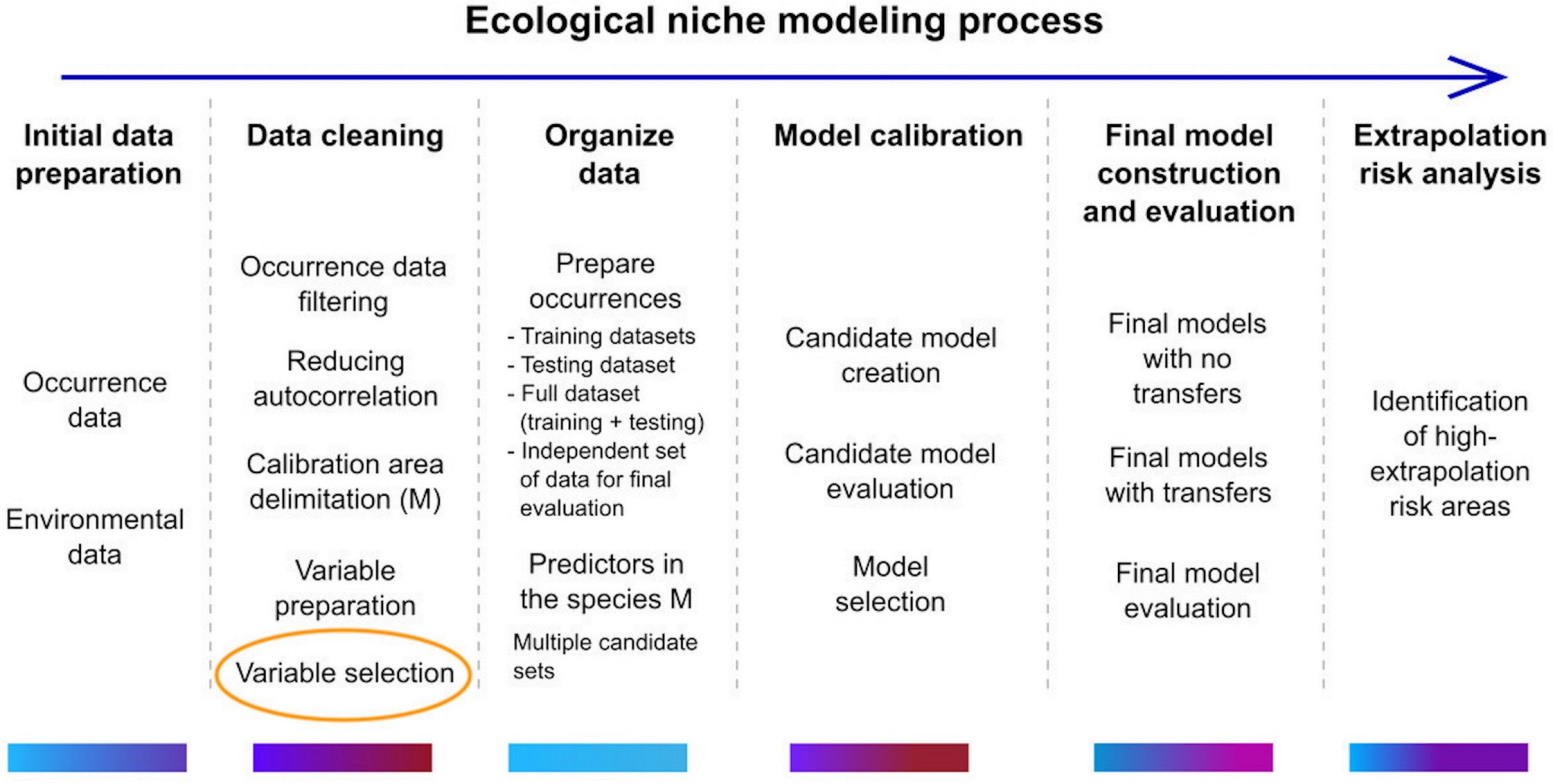
Ecological niche modeling process. Schematic description of the ecological niche modeling process. Color bars under each step reflect an approximate range of times that may be needed, ranging from low (blue) to high (red). Use of a Monte Carlo method to prescreen a large collection of predictors could support variable selection in the data cleaning step. Image provided by [22] and adapted for use here under a CC-BY license.

Cobos et al. [22] provide a useful framework for understanding where the results presented here might fit (Fig 4). The work of ecological niche modeling can be thought of as a multi-step process ranging from initial data preparation and cleaning, to model calibration, final model construction, model evaluation, and the assessment of extrapolation risk. Among the tasks associated with data cleaning, the selection of viable predictors is crucial, time-consuming, and the place where a means for rapid, automatic, preselection, however coarse, could improve the overall workflow, especially if it enabled exploration of a large universe of predictors.

The use of IPCC-class climate model outputs in efforts to assess the impacts of climate change on biodiversity and other ecosystem processes is growing. Exploring the potential of these massive data sets, expanded use of ensemble modeling, and the actual work of fitting models for the thousands of species scientists wish to study will require hundreds to thousands of projections [16, 25]. An improved capacity to use large environmental data sets in MaxEnt modeling would greatly benefit this work. We are encouraged to think that innovative use of Monte Carlo techniques might provide a helpful means of meeting this challenge.

## Conclusions

This small-scale, proof-of-concept study leaves many practical and theoretical questions unanswered. Preliminary results, however, suggest that a Monte Carlo approach might be an effective way to screen environmental predictors prior to final model construction that could be parallelized and scaled to large data sets, including externally-stored collections. This points to the possibility of near-real-time multiprocessor implementations that would enable broader and more exploratory use of global climate model outputs in environmental niche modeling and aid in the discovery of new predictors.

Next steps will focus on implementing a parallel, high-performance version of this capability in NASA’s science cloud, evaluating the method’s behavior using products generated by the Goddard Earth Observing System, Version 5 (GEOS-5) climate modeling system, extending stochasticity to feature class and regularization multiplier selection, and making various improvements to the algorithm, such as developing automatic stopping rules and developing better measures of variable importance. We also look forward to evaluating the method’s effectiveness in addressing research questions relating to climate change influences on Cassin’s Sparrow distribution.

## Supporting information

S1 Table Bioclim correlation analysis. Table shows values of Pearson correlation coefficient (r), Pearson coefficient of determination (r ²), and Variance Inflation Factor (VIF) for the Worldclim Bioclimatic variables for the study area [56]. Values of r > 0.8, r2 > 0.8, and VIF > 10.0 are highlighted and indicate highly correlated variables.
(PDF)

S2 Table ENMeval feature class and regularization multiplier scan. Table shows results from a comprehensive ENMeval scan of the 19 Bioclim variables over the study area [38, 59]. MaxEnt’s default feature class and regularization multiplier settings (LQHP, 1.0) resulted in the lowest AICc value and best overall model in the scan.
(PDF)

S1 File Study data and script. Compressed file folder containing the input data and R script used in the study.
(ZIP)
