# Supplementary material for: Toward a Monte Carlo approach to selecting climate variables in MaxEnt
Source: PLoS One. 2021 Mar 3;16(3):e0237208. doi: 10.1371/journal.pone.0237208 (PMC7928495; doi:10.1371/journal.pone.0237208)
Supplement: S1 Table — Table shows values of Pearson correlation coefficient (r), Pearson coefficient of determination (r ²), and Variance Inflation Factor (VIF) for the Worldclim Bioclimatic variables for the study area [56]. Values of r > 0.8, r2 > 0.8, and VIF > 10.0 are highlighted and indicate highly correlated variables. (PDF) [file pone.0237208.s001.pdf]

S1 Table. Bioclim Correlation Analysis.

		Bio 1	Bio 2	Bio 3	Bio 4	Bio 5	Bio 6	Bio 7	Bio 8	Bio 9	Bio 10	Bio 11	Bio 12	Bio 13	Bio 14	Bio 15	Bio 16	Bio 17	Bio 18	Bio 19
	r		-0.001	0.424	-0.437	**0.839**	**0.918**	-0.392	0.673	0.680	**0.897**	**0.950**	0.026	0.235	-0.294	0.522	0.176	-0.262	0.126	-0.090
Bio 1	r^2		0.000	0.180	0.191	0.704	**0.843**	0.154	0.453	0.462	0.804	**0.902**	0.001	0.055	0.086	0.272	0.031	0.069	0.016	0.008
	VIF		1.000	1.219	1.235	3.380	6.356	1.182	1.827	1.860	5.114	**10.165**	1.001	1.058	1.094	1.374	1.032	1.074	1.016	1.008
	r			0.558	0.080	0.284	-0.203	0.481	0.148	0.165	0.045	-0.019	-0.272	-0.055	-0.410	0.351	-0.029	-0.418	0.029	-0.216
Bio 2	r^2			0.311	0.006	0.081	0.041	0.232	0.022	0.027	0.002	0.000	0.074	0.003	0.168	0.123	0.001	0.175	0.001	0.047
	VIF			1.451	1.006	1.088	1.043	1.302	1.022	1.028	1.002	1.000	1.080	1.003	1.203	1.140	1.001	1.212	1.001	1.049
	r				-0.697	0.234	0.485	-0.383	0.250	0.522	0.135	0.585	-0.029	0.305	-0.420	0.690	0.311	-0.401	0.272	0.027
Bio 3	r^2				0.486	0.055	0.235	0.147	0.062	0.273	0.018	0.342	0.001	0.093	0.177	0.476	0.097	0.161	0.074	0.001
	VIF				1.946	1.058	1.307	1.172	1.067	1.375	1.019	1.519	1.001	1.103	1.214	1.909	1.107	1.192	1.080	1.001
	r					0.065	-0.716	**0.910**	-0.136	-0.520	0.002	-0.693	-0.040	-0.320	0.275	-0.533	-0.300	0.248	-0.224	-0.131
Bio 4	r^2					0.004	0.513	**0.829**	0.019	0.270	0.000	0.480	0.002	0.103	0.076	0.284	0.090	0.061	0.050	0.017
	VIF					1.004	2.052	5.833	1.019	1.370	1.000	1.923	1.002	1.114	1.082	1.396	1.099	1.065	1.053	1.018
	r						0.565	0.165	0.665	0.506	**0.968**	0.654	-0.074	0.061	-0.289	0.372	0.018	-0.272	0.013	-0.210
Bio 5	r^2						0.319	0.027	0.442	0.256	**0.937**	0.427	0.006	0.004	0.084	0.138	0.000	0.074	0.000	0.044
	VIF						1.469	1.028	1.793	1.344	**15.822**	1.746	1.006	1.004	1.091	1.161	1.000	1.080	1.000	1.046
	r							-0.721	0.522	0.690	0.671	**0.981**	0.069	0.297	-0.266	0.538	0.242	-0.232	0.164	0.027
Bio 6	r^2							0.519	0.273	0.475	0.451	**0.963**	0.005	0.088	0.071	0.290	0.059	0.054	0.027	0.001
	VIF							2.081	1.375	1.906	1.820	**26.724**	1.005	1.097	1.076	1.408	1.062	1.057	1.028	1.001
	r								-0.066	-0.399	0.011	-0.624	-0.145	-0.304	0.075	-0.331	-0.275	0.048	-0.186	-0.209
Bio 7	r^2								0.004	0.159	0.000	0.389	0.021	0.092	0.006	0.109	0.075	0.002	0.035	0.044
	VIF								1.004	1.190	1.000	1.636	1.022	1.102	1.006	1.123	1.082	1.002	1.036	1.046
	r									0.231	0.666	0.571	0.147	0.303	-0.165	0.482	0.262	-0.162	0.327	-0.267
Bio 8	r^2									0.053	0.443	0.327	0.022	0.092	0.027	0.232	0.069	0.026	0.107	0.072
	VIF									1.056	1.795	1.485	1.022	1.101	1.028	1.302	1.074	1.027	1.119	1.077
	r										0.522	0.744	-0.258	-0.011	-0.440	0.367	-0.059	-0.386	-0.150	0.144
Bio 9	r^2										0.272	0.553	0.066	0.000	0.194	0.134	0.003	0.149	0.023	0.021
	VIF										1.374	2.239	1.071	1.000	1.241	1.155	1.003	1.176	1.023	1.021
	r											0.719	-0.019	0.082	-0.216	0.321	0.027	-0.194	0.005	-0.159
Bio 10	r^2											0.517	0.000	0.007	0.047	0.103	0.001	0.038	0.000	0.025
	VIF											2.071	1.000	1.007	1.049	1.115	1.001	1.039	1.000	1.026
	r												0.011	0.279	-0.349	0.600	0.226	-0.314	0.157	-0.018
Bio 11	r^2												0.000	0.078	0.122	0.360	0.051	0.099	0.025	0.000
	VIF												1.000	1.085	1.139	1.564	1.054	1.109	1.025	1.000
	r													**0.863**	0.631	0.078	**0.861**	0.668	**0.813**	0.555
Bio 12	r^2													0.745	0.399	0.006	0.741	0.447	0.661	0.308
	VIF													3.918	1.663	1.006	3.859	1.807	2.948	1.445
	r														0.235	0.490	**0.986**	0.275	**0.935**	0.394
Bio 13	r^2														0.055	0.240	**0.971**	0.075	**0.875**	0.155
	VIF														1.058	1.315	**34.986**	1.082	8.000	1.184
	r															-0.557	0.216	**0.983**	0.200	0.483
Bio 14	r^2															0.310	0.047	**0.966**	0.040	0.233
	VIF															1.450	1.049	**29.152**	1.042	1.303
	r																0.505	-0.554	0.473	-0.169
Bio 15	r2																0.255	0.307	0.224	0.029
	VIF																1.342	1.444	1.288	1.030
	r																	0.248	**0.950**	0.373
Bio 16	r^2																	0.062	**0.902**	0.139
	VIF																	1.066	**10.171**	1.162
	r																		0.224	0.550
Bio 17	r^2																		0.050	0.302
	VIF																		1.053	1.434
	r																			0.214
Bio 18	r^2																			0.046
	VIF																			1.048
	r																			
Bio 19	r^2																			
	VIF																			
