# Supplementary material for: Toward a Monte Carlo approach to selecting climate variables in MaxEnt
Source: PLoS One. 2021 Mar 3;16(3):e0237208. doi: 10.1371/journal.pone.0237208 (PMC7928495; doi:10.1371/journal.pone.0237208)
Supplement: S2 Table — Table shows results from a comprehensive ENMeval scan of the 19 Bioclim variables over the study area [38, 59]. MaxEnt’s default feature class and regularization multiplier settings (LQHP, 1.0) resulted in the lowest AICc value and best overall model in the scan. (PDF) [file pone.0237208.s002.pdf]

S2 Table. ENMeval Feature Class and Regularization Multiplier Scan.

	settings	features	rm	train.AUC	avg.test.AUC	var.test.AUC	avg.diff.AUC	var.diff.AUC	avg.test.orMTP	var.test.orMTP	avg.test.or10pct	var.test.or10pct	AICc	delta.AICc	w.AIC	parameters
1	L_0.5	L	0.5	0.5554	0.522482743	0.009828229	0.039713398	0.009681591	0.003278689	4.78E-05	0.116693989	0.00180239	12909.84862	378.1636922	2.74E-83	14
2	LQ_0.5	LQ	0.5	0.6081	0.580776749	0.009491528	0.034170297	0.007958198	0.003278689	4.78E-05	0.124754098	0.001256801	12630.90673	99.22180154	1.02E-22	22
3	H_0.5	H	0.5	0.6534	0.574461093	0.008691308	0.082685676	0.009088654	0.024617486	0.00055193	0.149398907	0.002526879	15060.43947	2528.754543	0	84
4	LQH_0.5	LQH	0.5	0.6534	0.574463716	0.008691307	0.082757013	0.009136991	0.022978142	0.000429601	0.152677596	0.003098609	15060.43947	2528.754543	0	84
5	LQHP_0.5	LQHP	0.5	0.6534	0.575431619	0.008586036	0.082660917	0.00878169	0.022978142	0.000429601	0.156010929	0.003006709	15060.43947	2528.754543	0	84
6	LQHPT_0.5	LQHPT	0.5	0.6534	0.575431619	0.008586036	0.082660917	0.00878169	0.022978142	0.000429601	0.156010929	0.003006709	15060.43947	2528.754543	0	84
7	L_1	L	1	0.5316	0.519117989	0.0114877	0.025323948	0.007198028	0.001639344	2.69E-05	0.108360656	0.001322494	12954.43936	422.7544255	5.69E-93	5
8	LQ_1	LQ	1	0.5386	0.519075697	0.009618943	0.026544631	0.007676098	0.001639344	2.69E-05	0.110054645	0.000551305	12923.85092	392.1659925	2.50E-86	8
9	H_1	H	1	0.6224	0.585894301	0.008094387	0.041890321	0.006606099	0.009863388	0.000131792	0.134535519	0.001643644	12553.31931	21.63437591	7.20E-06	43
10	LQH_1	LQH	1	0.6224	0.582014587	0.008516185	0.045654071	0.007311879	0.009863388	0.000131792	0.132923497	0.00144494	12553.31931	21.63437591	7.20E-06	43
11	LQHP_1	**LQHP**	**1**	0.6258	0.588140669	0.006360179	0.041744259	0.005955404	0.006584699	7.23E-05	0.139590164	0.000676946	12531.68493	0	0.359269081	53
12	LQHPT_1	LQHPT	1	0.6258	0.588140669	0.006360179	0.041744259	0.005955404	0.006584699	7.23E-05	0.139590164	0.000676946	12531.68493	0	0.359269081	53
13	L_1.5	L	1.5	0.5325	0.516751071	0.010887471	0.026851351	0.007497633	0.001639344	2.69E-05	0.110027322	0.001319306	12947.14068	415.4557454	2.19E-91	6
14	LQ_1.5	LQ	1.5	0.5633	0.531828973	0.010733057	0.037460895	0.009162646	0.004918033	6.27E-05	0.118360656	0.001778375	12889.56966	357.8847293	6.95E-79	13
15	H_1.5	H	1.5	0.6298	0.581878941	0.009588108	0.053792812	0.008996582	0.009836066	0.000191108	0.129617486	0.001743378	12559.71114	28.02620478	2.95E-07	65
16	LQH_1.5	LQH	1.5	0.63	0.580377563	0.009796933	0.055760035	0.009257052	0.009836066	0.000191108	0.132978142	0.001154277	12559.94757	28.26263892	2.62E-07	67
17	LQHP_1.5	LQHP	1.5	0.6352	0.588544631	0.00763299	0.050983237	0.007649936	0.008224044	0.000134878	0.142868852	0.0012007	12542.29129	10.6063592	0.001787632	82
18	LQHPT_1.5	LQHPT	1.5	0.6352	0.588544631	0.00763299	0.050983237	0.007649936	0.008224044	0.000134878	0.142868852	0.0012007	12542.29129	10.6063592	0.001787632	82
19	L_2	L	2	0.5355	0.512876272	0.008943096	0.029798219	0.007475742	0.003278689	4.78E-05	0.110081967	0.00139778	12943.07993	411.3949973	1.67E-90	9
20	LQ_2	LQ	2	0.594	0.561860232	0.012019685	0.038344021	0.010739998	0.009836066	0.000131386	0.134726776	0.001632086	12794.9618	263.2768695	2.43E-58	19
21	H_2	H	2	0.6374	0.5817565	0.011049622	0.060089436	0.011949594	0.011502732	0.000182455	0.13295082	0.001746514	12630.5671	98.88216803	1.21E-22	105
22	LQH_2	LQH	2	0.6373	0.579805227	0.010685547	0.062316943	0.011881113	0.013142077	0.000286868	0.134562842	0.001823671	12614.22909	82.54415771	4.28E-19	101
23	LQHP_2	LQHP	2	0.6416	0.589332019	0.008350795	0.056263105	0.009613704	0.006557377	0.000131386	0.142868852	0.00173819	12593.83914	62.15420454	1.15E-14	104
24	LQHPT_2	LQHPT	2	0.6416	0.589332019	0.008350795	0.056263105	0.009613704	0.006557377	0.000131386	0.142868852	0.00173819	12593.83914	62.15420454	1.15E-14	104
25	L_2.5	L	2.5	0.5314	0.51986548	0.01098761	0.024121645	0.006649944	0.001639344	2.69E-05	0.105081967	0.001204246	12963.49428	431.8093446	6.15E-95	4
26	LQ_2.5	LQ	2.5	0.5384	0.518569079	0.010109175	0.026522695	0.008133702	0.001639344	2.69E-05	0.108415301	0.000799944	12938.19492	406.5099897	1.92E-89	7
27	H_2.5	H	2.5	0.6145	0.583801325	0.008053506	0.034390974	0.004395374	0.008224044	0.000134878	0.118114754	0.002006099	12585.27137	53.58643851	8.30E-13	37
28	LQH_2.5	LQH	2.5	0.6139	0.580078851	0.006600679	0.036158993	0.004856193	0.009863388	0.000251234	0.123032787	0.001673751	12599.58653	67.90159495	6.47E-16	43
29	LQHP_2.5	LQHP	2.5	0.6195	0.588158124	0.006464785	0.036226748	0.004794228	0.006584699	7.23E-05	0.136284153	0.001016334	12533.58505	1.900119329	0.138935802	34
30	LQHPT_2.5	LQHPT	2.5	0.6195	0.588158124	0.006464785	0.036226748	0.004794228	0.006584699	7.23E-05	0.136284153	0.001016334	12533.58505	1.900119329	0.138935802	34
31	L_3	L	3	0.5316	0.519156093	0.010990881	0.023886928	0.006626526	0.001639344	2.69E-05	0.11	0.001850428	12973.87437	442.1894377	3.43E-97	4
32	LQ_3	LQ	3	0.5348	0.516937836	0.010543841	0.026394864	0.008405782	0.001639344	2.69E-05	0.108387978	0.00073175	12959.23623	427.5512941	5.17E-94	8
33	H_3	H	3	0.6006	0.579181316	0.010522213	0.033655611	0.004797368	0.009863388	0.000251234	0.109918033	0.002082243	12610.01893	78.33399674	3.51E-18	29
34	LQH_3	LQH	3	0.6005	0.573521337	0.007391711	0.03517167	0.00504392	0.009863388	0.000251234	0.111557377	0.002007293	12628.08696	96.40202637	4.19E-22	37
35	LQHP_3	LQHP	3	0.6154	0.585193601	0.006519249	0.035336493	0.004353288	0.004945355	6.34E-05	0.13136612	0.000956015	12569.4178	37.73287106	2.30E-09	33
36	LQHPT_3	LQHPT	3	0.6154	0.585193601	0.006519249	0.035336493	0.004353288	0.004945355	6.34E-05	0.13136612	0.000956015	12569.4178	37.73287106	2.30E-09	33
37	L_3.5	L	3.5	0.5281	0.517386051	0.011007286	0.023488653	0.006758943	0.001639344	2.69E-05	0.119863388	0.001972058	12988.16837	456.48344	2.70E-100	5
38	LQ_3.5	LQ	3.5	0.5313	0.515441617	0.010774738	0.025728812	0.008348468	0.001639344	2.69E-05	0.113306011	0.000625148	12970.63517	438.9502417	1.73E-96	7
39	H_3.5	H	3.5	0.5957	0.57851574	0.011628851	0.031165313	0.005295744	0.008224044	0.000134878	0.111557377	0.001648967	12648.28764	116.6027045	1.72E-26	30
40	LQH_3.5	LQH	3.5	0.5957	0.571555574	0.007249283	0.033237646	0.004724316	0.008224044	0.000134878	0.114836066	0.001540872	12648.28764	116.6027045	1.72E-26	30
41	LQHP_3.5	LQHP	3.5	0.6076	0.580722522	0.005937593	0.033587728	0.00425961	0.006584699	7.23E-05	0.128060109	0.001296712	12598.45908	66.77414938	1.14E-15	25
42	LQHPT_3.5	LQHPT	3.5	0.6076	0.580722522	0.005937593	0.033587728	0.00425961	0.006584699	7.23E-05	0.128060109	0.001296712	12598.45908	66.77414938	1.14E-15	25
43	L_4	L	4	0.5268	0.517608279	0.011173645	0.022851529	0.006605521	0.001639344	2.69E-05	0.118224044	0.002136988	12994.99745	463.3125205	8.88E-102	4
44	LQ_4	LQ	4	0.5291	0.516577989	0.011450211	0.024419781	0.008059042	0.001639344	2.69E-05	0.118224044	0.001121729	12977.86017	446.1752352	4.67E-98	5
45	H_4	H	4	0.5938	0.57368697	0.011127439	0.030878127	0.006216059	0.008224044	0.000134878	0.11647541	0.001985495	12682.34061	150.6556798	6.93E-34	28
46	LQH_4	LQH	4	0.5938	0.568205911	0.007831907	0.032160881	0.005286664	0.008224044	0.000134878	0.11647541	0.001866053	12681.68317	149.9982406	9.63E-34	28
47	LQHP_4	LQHP	4	0.5996	0.577382056	0.005269511	0.030055737	0.003849446	0.008224044	0.000134878	0.12147541	0.001616322	12643.47975	111.794819	1.90E-25	25
48	LQHPT_4	LQHPT	4	0.5996	0.577382056	0.005269511	0.030055737	0.003849446	0.008224044	0.000134878	0.12147541	0.001616322	12643.47975	111.794819	1.90E-25	25
